# Supplementary figures and images for: Genomes of the autonomous parvovirus minute virus of mice induce replication stress through RPA exhaustion
Source: PLoS Pathog. 2023 May 30;19(5):e1011203. doi: 10.1371/journal.ppat.1011203 (PMC10256180; doi:10.1371/journal.ppat.1011203)

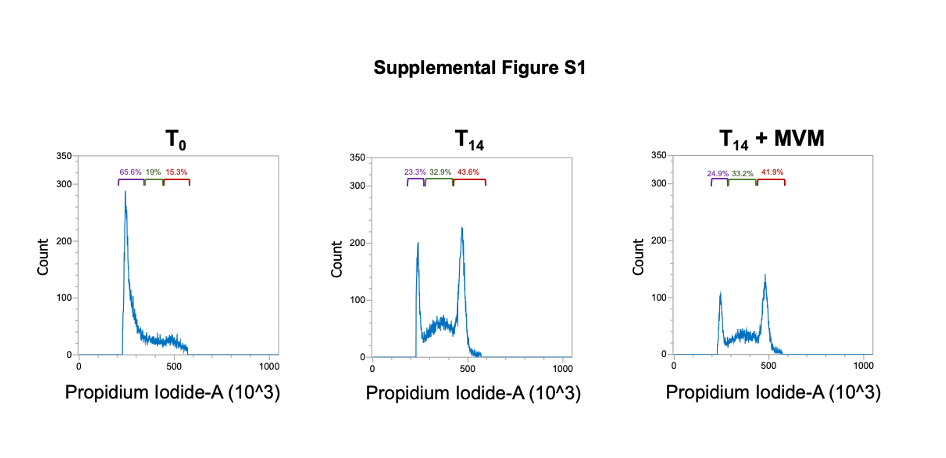

Supplement: S1 Fig — A9 cells were plated in Isoleucine-deficient media for 36–42 hours (labelled as T0), before being released into complete DMEM media and infected (see Materials and Methods for details). Cells were harvested at 0 hours post release or 14 hours post release and processed for cell cycle analysis by propidium iodide staining. Cells enter S phase at 12 hours post-release, and are not impacted by the presence or absence of MVM (middle and right panels). (TIFF) [file ppat.1011203.s001.tiff]

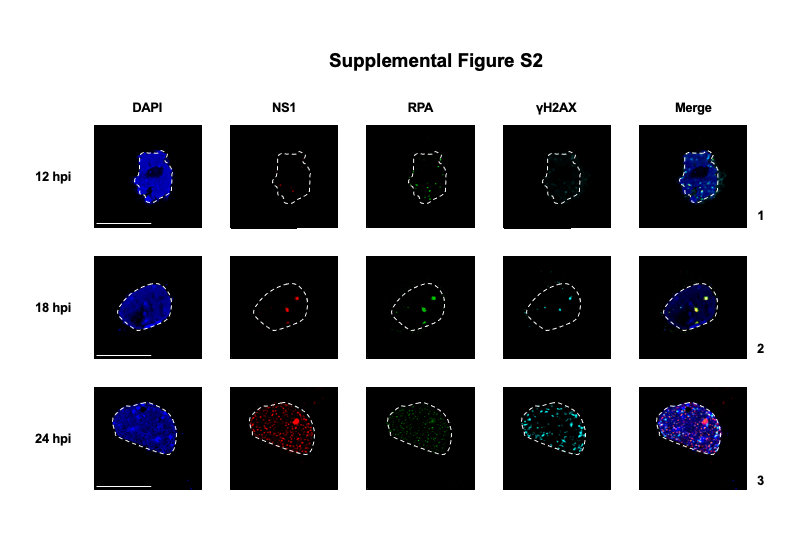

Supplement: S2 Fig — MVM infected A9 cells at 12 hpi (panel 1), 18 hpi (panel 2) and 24 hpi (panel 3) were processed for 4-color imaging. Nuclear borders were demarcated by white dashed lines and scale bars represent 10 microns. (TIFF) [file ppat.1011203.s002.tiff]

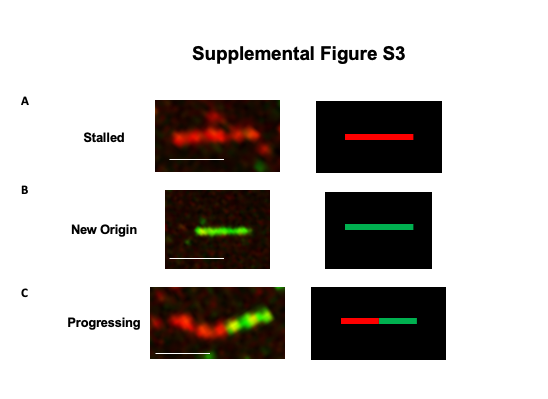

Supplement: S3 Fig — Representative images of replication fibers in A9 cells that are (A) stalled, (B) new origin and (C) progressing, with their corresponding schematic for categorization shown on the right of the respective panel. Scale bars represent 4 micrometers. (TIFF) [file ppat.1011203.s003.tiff]

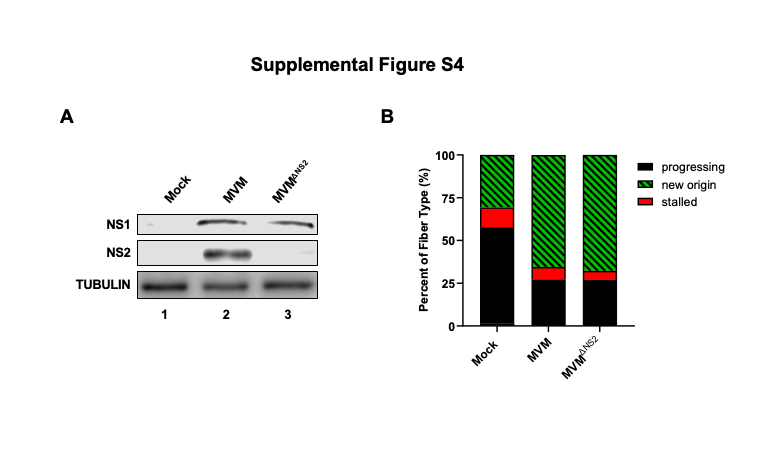

Supplement: S4 Fig — (A) U2OS cells were infected with wild-type MVMp or MVMΔNS2 for 24 hours, and processed for NS1, NS2 and Tubulin levels by immunoblot. (B) Categorization of replication forks in U2OS cells infected with wild-type MVMp or MVMΔNS2 for 24 hours. (TIFF) [file ppat.1011203.s004.tiff]

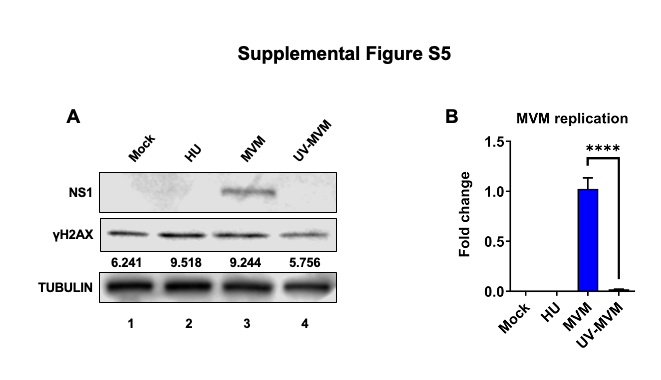

Supplement: S5 Fig — (A) Immunoblots showing inactivation of UV-MVM by monitoring NS1 levels during infection of A9 cells for 24 hours. Verification of lack of induction of cellular γH2AX upon infection of A9 cells with UV-MVM for 24 hpi. The respective band intensities are shown below the sample (B) Taqman qPCR analysis of MVMp with the probe directed against the plus strand of the MVM genome, showing UV-MVM is made up of single stranded DNA at 18 hpi and therefore cannot be detected, **** represents P ≤ 0.0001. (TIFF) [file ppat.1011203.s005.tiff]

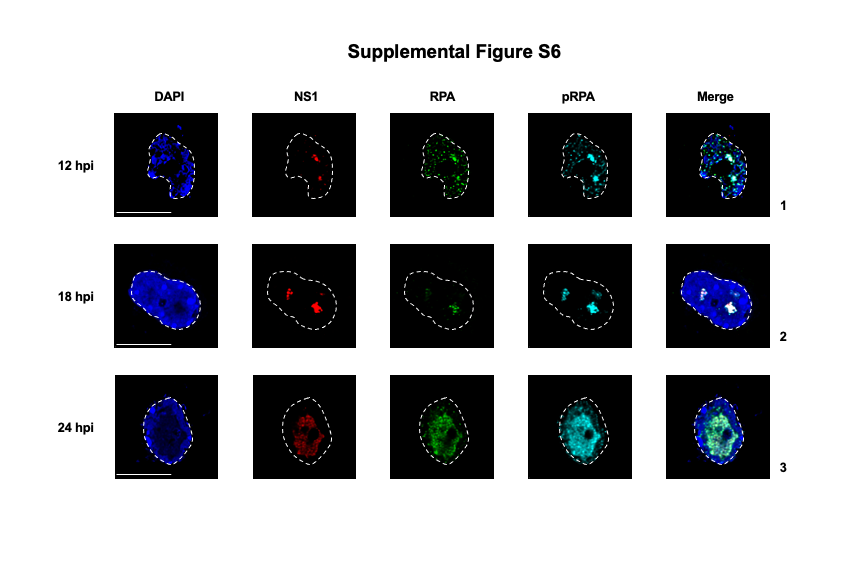

Supplement: S6 Fig — MVM infected A9 cells at 12 hpi (panel 1), 18 hpi (panel 2) and 24 hpi (panel 3) were processed for 4-color imaging for the indicated proteins. Nuclear borders were demarcated by white dashed lines and scale bars represent 10 microns. (TIFF) [file ppat.1011203.s006.tiff]

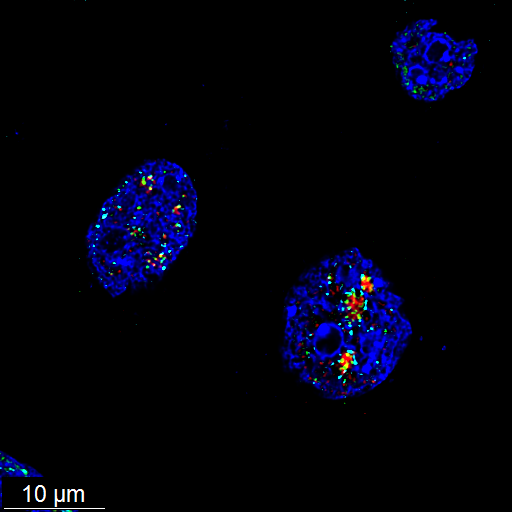

Supplement: S1 File — Supplemental.zip file of all DNA fiber assay measurements. (ZIP) [file ppat.1011203.s007.zip › RPA Paper Information/Exhaustion Paper Images/02.07.23 MVM A9 4 colors_Series003_Lng_z07.tif]

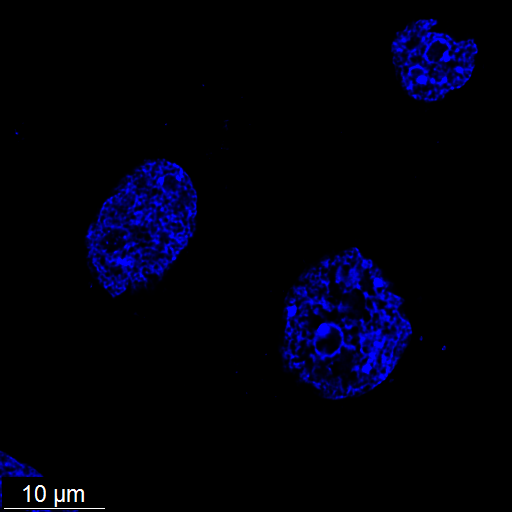

Supplement: S1 File — Supplemental.zip file of all DNA fiber assay measurements. (ZIP) [file ppat.1011203.s007.zip › RPA Paper Information/Exhaustion Paper Images/02.07.23 MVM A9 4 colors_Series003_Lng_z07_ch00.tif]

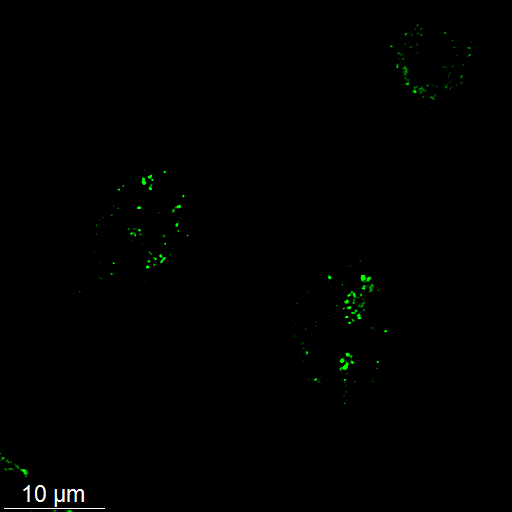

Supplement: S1 File — Supplemental.zip file of all DNA fiber assay measurements. (ZIP) [file ppat.1011203.s007.zip › RPA Paper Information/Exhaustion Paper Images/02.07.23 MVM A9 4 colors_Series003_Lng_z07_ch01.tif]

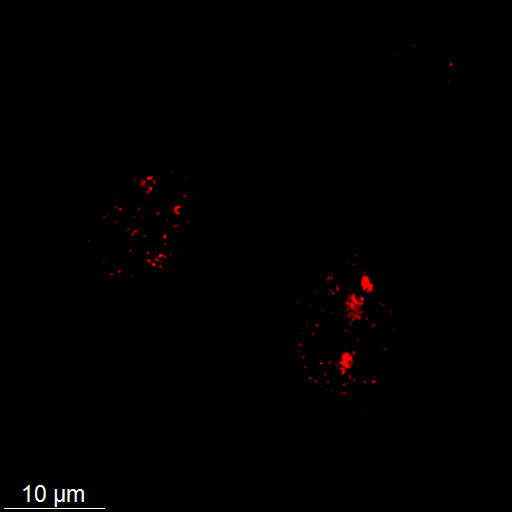

Supplement: S1 File — Supplemental.zip file of all DNA fiber assay measurements. (ZIP) [file ppat.1011203.s007.zip › RPA Paper Information/Exhaustion Paper Images/02.07.23 MVM A9 4 colors_Series003_Lng_z07_ch02.tif]

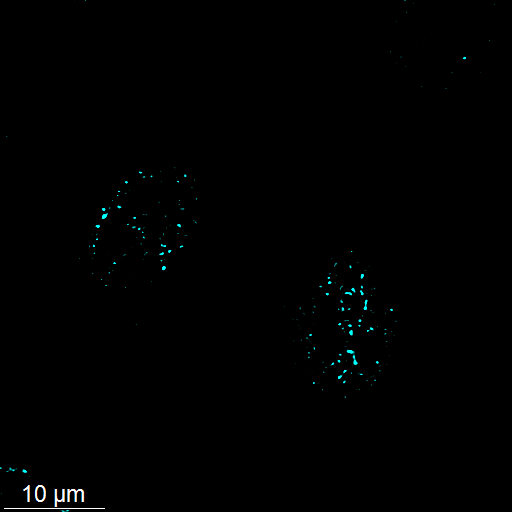

Supplement: S1 File — Supplemental.zip file of all DNA fiber assay measurements. (ZIP) [file ppat.1011203.s007.zip › RPA Paper Information/Exhaustion Paper Images/02.07.23 MVM A9 4 colors_Series003_Lng_z07_ch03.tif]

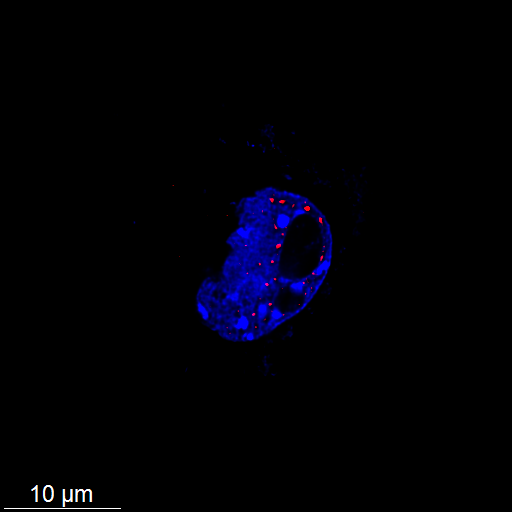

Supplement: S1 File — Supplemental.zip file of all DNA fiber assay measurements. (ZIP) [file ppat.1011203.s007.zip › RPA Paper Information/Exhaustion Paper Images/10.12.22 MVM iCDC7_Series003_Lng_z03.tif]

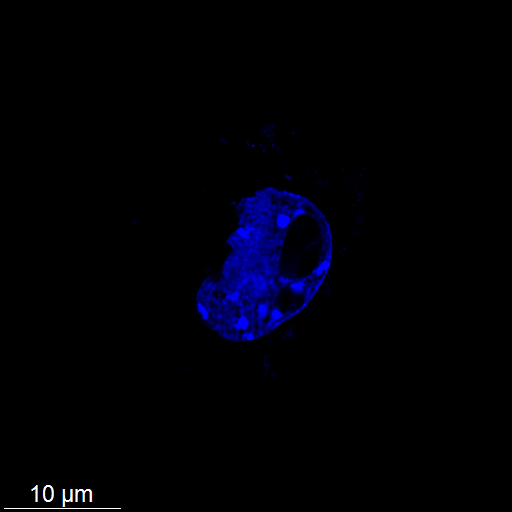

Supplement: S1 File — Supplemental.zip file of all DNA fiber assay measurements. (ZIP) [file ppat.1011203.s007.zip › RPA Paper Information/Exhaustion Paper Images/10.12.22 MVM iCDC7_Series003_Lng_z03_ch00.tif]

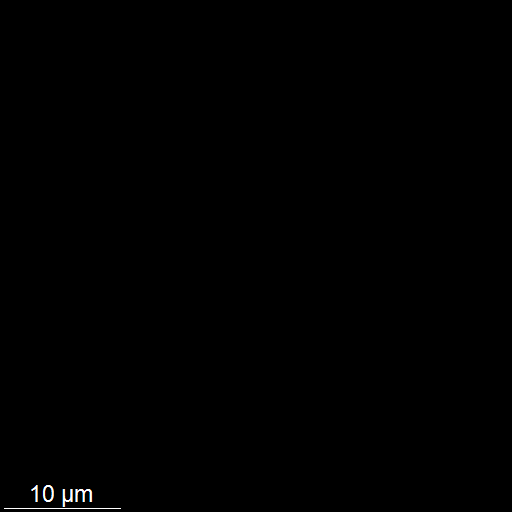

Supplement: S1 File — Supplemental.zip file of all DNA fiber assay measurements. (ZIP) [file ppat.1011203.s007.zip › RPA Paper Information/Exhaustion Paper Images/10.12.22 MVM iCDC7_Series003_Lng_z03_ch01.tif]

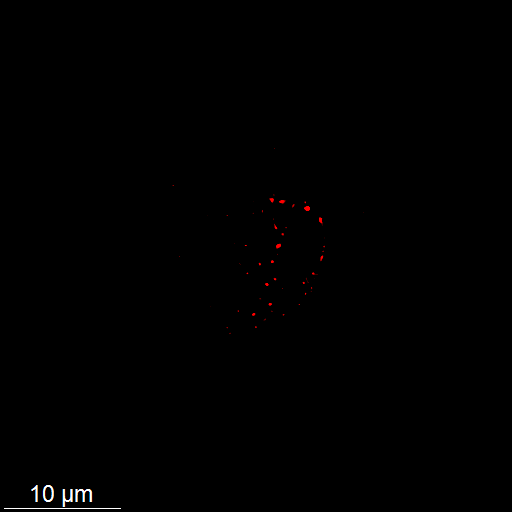

Supplement: S1 File — Supplemental.zip file of all DNA fiber assay measurements. (ZIP) [file ppat.1011203.s007.zip › RPA Paper Information/Exhaustion Paper Images/10.12.22 MVM iCDC7_Series003_Lng_z03_ch02.tif]

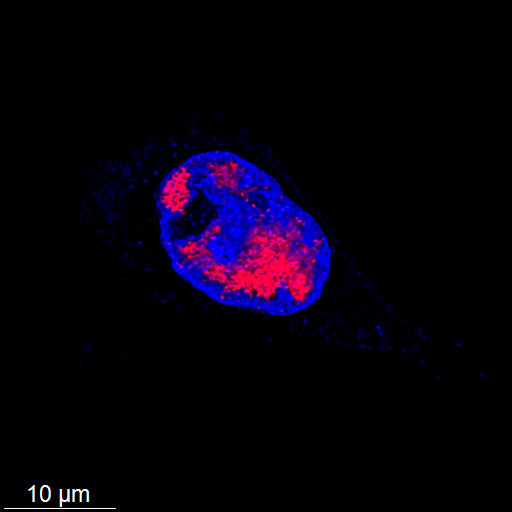

Supplement: S1 File — Supplemental.zip file of all DNA fiber assay measurements. (ZIP) [file ppat.1011203.s007.zip › RPA Paper Information/Exhaustion Paper Images/10.12.22 MVM Mock_Series004_Lng_z04.tif]

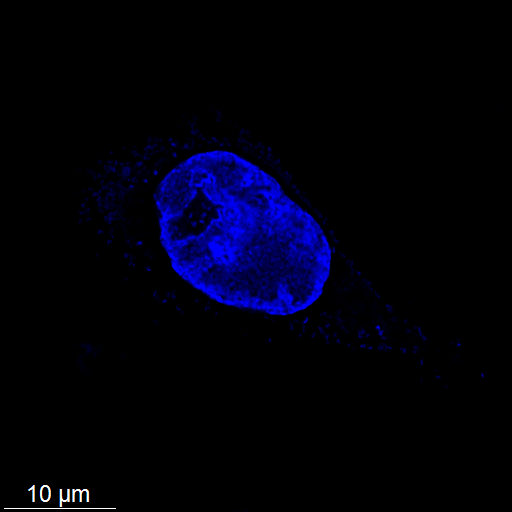

Supplement: S1 File — Supplemental.zip file of all DNA fiber assay measurements. (ZIP) [file ppat.1011203.s007.zip › RPA Paper Information/Exhaustion Paper Images/10.12.22 MVM Mock_Series004_Lng_z04_ch00.tif]

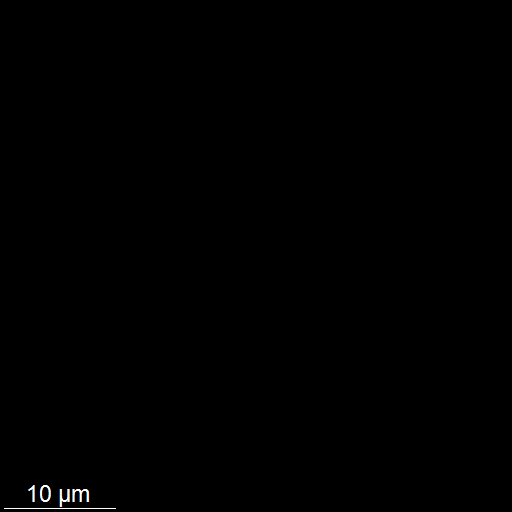

Supplement: S1 File — Supplemental.zip file of all DNA fiber assay measurements. (ZIP) [file ppat.1011203.s007.zip › RPA Paper Information/Exhaustion Paper Images/10.12.22 MVM Mock_Series004_Lng_z04_ch01.tif]

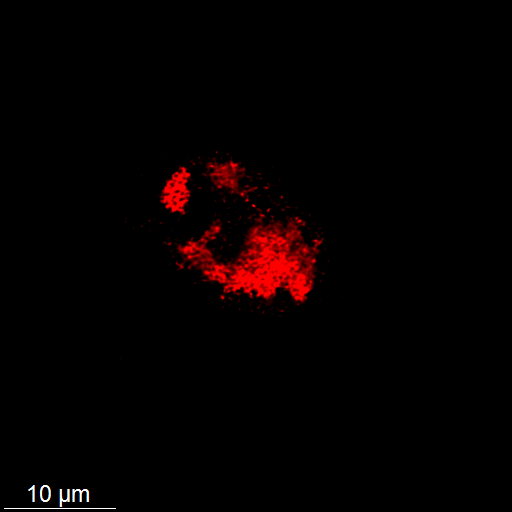

Supplement: S1 File — Supplemental.zip file of all DNA fiber assay measurements. (ZIP) [file ppat.1011203.s007.zip › RPA Paper Information/Exhaustion Paper Images/10.12.22 MVM Mock_Series004_Lng_z04_ch02.tif]

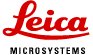

Supplement: S1 File — Supplemental.zip file of all DNA fiber assay measurements. (ZIP) [file ppat.1011203.s007.zip › RPA Paper Information/Exhaustion Paper Images/MetaData/LeicaLogo.jpg]

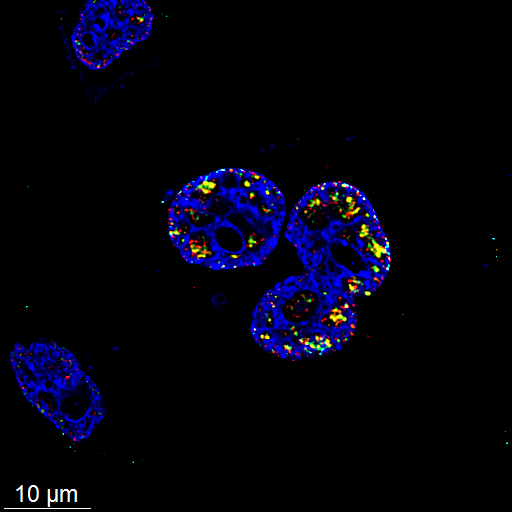

Supplement: S1 File — Supplemental.zip file of all DNA fiber assay measurements. (ZIP) [file ppat.1011203.s007.zip › RPA Paper Information/Exhaustion Paper Images/MVM infected A9 NS1_568 RPA_488 gH2AX_633_Series004_Lng_z09.tif]

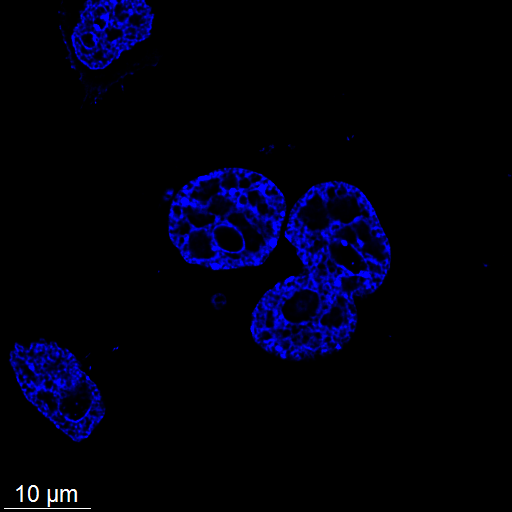

Supplement: S1 File — Supplemental.zip file of all DNA fiber assay measurements. (ZIP) [file ppat.1011203.s007.zip › RPA Paper Information/Exhaustion Paper Images/MVM infected A9 NS1_568 RPA_488 gH2AX_633_Series004_Lng_z09_ch00.tif]

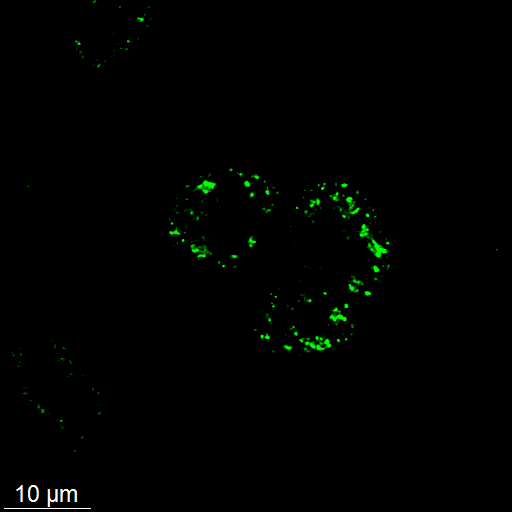

Supplement: S1 File — Supplemental.zip file of all DNA fiber assay measurements. (ZIP) [file ppat.1011203.s007.zip › RPA Paper Information/Exhaustion Paper Images/MVM infected A9 NS1_568 RPA_488 gH2AX_633_Series004_Lng_z09_ch01.tif]

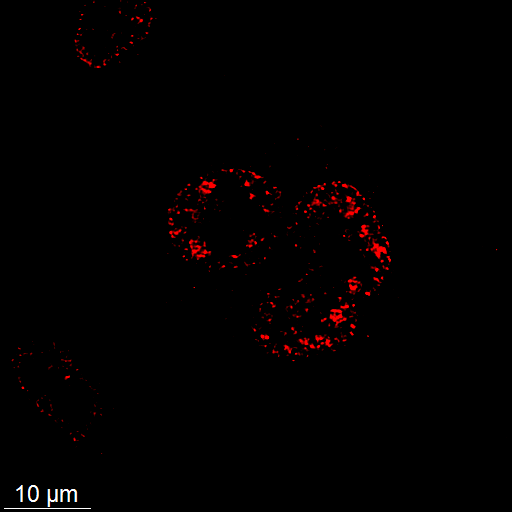

Supplement: S1 File — Supplemental.zip file of all DNA fiber assay measurements. (ZIP) [file ppat.1011203.s007.zip › RPA Paper Information/Exhaustion Paper Images/MVM infected A9 NS1_568 RPA_488 gH2AX_633_Series004_Lng_z09_ch02.tif]

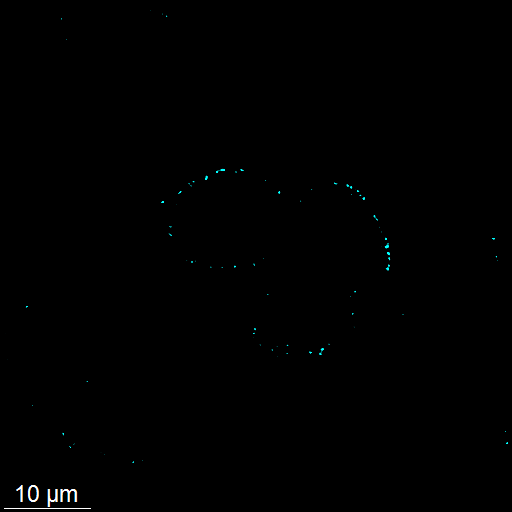

Supplement: S1 File — Supplemental.zip file of all DNA fiber assay measurements. (ZIP) [file ppat.1011203.s007.zip › RPA Paper Information/Exhaustion Paper Images/MVM infected A9 NS1_568 RPA_488 gH2AX_633_Series004_Lng_z09_ch03.tif]

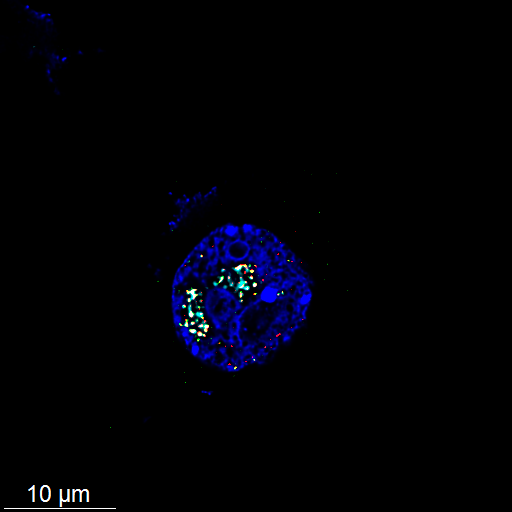

Supplement: S1 File — Supplemental.zip file of all DNA fiber assay measurements. (ZIP) [file ppat.1011203.s007.zip › RPA Paper Information/Exhaustion Paper Images/MVM infected A9 NS1_568 RPA_488 phosphoRPA_633_Series001_Lng_z09.tif]

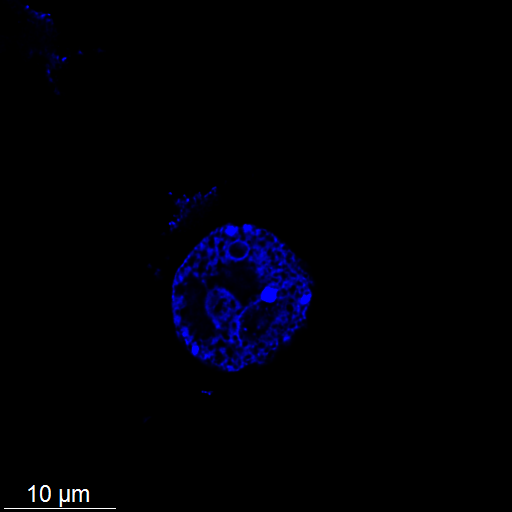

Supplement: S1 File — Supplemental.zip file of all DNA fiber assay measurements. (ZIP) [file ppat.1011203.s007.zip › RPA Paper Information/Exhaustion Paper Images/MVM infected A9 NS1_568 RPA_488 phosphoRPA_633_Series001_Lng_z09_ch00.tif]

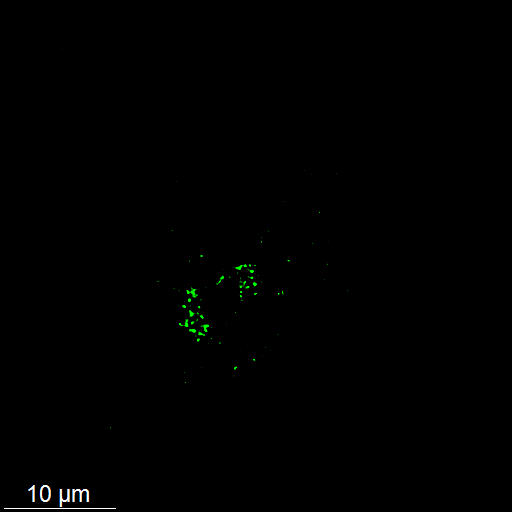

Supplement: S1 File — Supplemental.zip file of all DNA fiber assay measurements. (ZIP) [file ppat.1011203.s007.zip › RPA Paper Information/Exhaustion Paper Images/MVM infected A9 NS1_568 RPA_488 phosphoRPA_633_Series001_Lng_z09_ch01.tif]

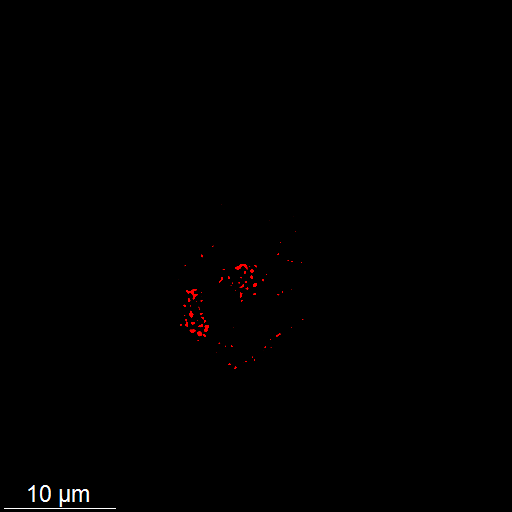

Supplement: S1 File — Supplemental.zip file of all DNA fiber assay measurements. (ZIP) [file ppat.1011203.s007.zip › RPA Paper Information/Exhaustion Paper Images/MVM infected A9 NS1_568 RPA_488 phosphoRPA_633_Series001_Lng_z09_ch02.tif]

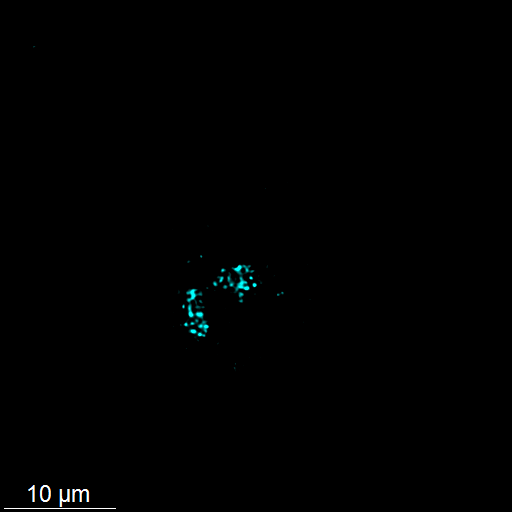

Supplement: S1 File — Supplemental.zip file of all DNA fiber assay measurements. (ZIP) [file ppat.1011203.s007.zip › RPA Paper Information/Exhaustion Paper Images/MVM infected A9 NS1_568 RPA_488 phosphoRPA_633_Series001_Lng_z09_ch03.tif]
