# Supplementary material for: Genomes of the autonomous parvovirus minute virus of mice induce replication stress through RPA exhaustion
Source: PLoS Pathog. 2023 May 30;19(5):e1011203. doi: 10.1371/journal.ppat.1011203 (PMC10256180; doi:10.1371/journal.ppat.1011203)
Supplement: Attachment — Submitted filename: CoverLetter_Point_by_point_Haubold_2023.docx [file ppat.1011203.s008.docx]

16 May 2023

Dear Colleagues,

Thank you for providing the reviews of our PLOS Pathogens manuscript, titled “Genomes of the Autonomous Parvovirus Minute Virus of Mice Induce Replication Stress Through RPA Exhaustion” (PPATHOGENS-D-23-00282). We were pleased that the reviewers and editors found our work novel and significant. As requested, we have addressed all the general and specific points raised by each reviewer (major changes highlighted in yellow in the revised text). Our responses and revisions to the reviewers’ comments are detailed below and the line callouts refer to the marked-up manuscript.

**REVIEWER 1**

We thank Reviewer 1, who found our studies to be “both interesting and novel” for their evaluative comments and recommendations to improve the manuscript. We have performed the suggested experiments and addressed their concerns as detailed below.

**P1.** The authors performed single-molecule DNA Fiber Analysis (DFA) by IdU and CIdU pulsing in para-synchronized cells, showing a shortening of labelled DNA fibers upon MVM infection.

(A) The efficient synchronization should be shown
(B) Fig. 1B: HU was used as pos. ctrl. (HU-induced replication stress). However, no statistics is shown from the 2way ANOVA multiple comparison, even though the difference is significant, which is not that obvious by simply looking at the medians. The same holds true for Fig. 1C.
(C) The authors state in the figure 1 legend that statistical analysis for Fig. 1B and 1C was performed using Mann Whitney Wilcoxon test. However, statistical analysis in Fig. 1B was done by 2way ANOVA multiple comparison (see RPA Paper Information).

**Response:** Thank you for the suggestions. Our responses are below:

(A) We have performed flow cytometry to validate the parasynchronization of A9 cells, which has been added as Supplemental Figure S1. These studies are also consistent with A9 synchronization protocols that have been previously published (Adeyemi et al., 2010; Cotmore and Tattersall, 1987; Clemens and Pintel, 1988).
(B) We have added the statistical tests in Figs. 1B and 1C showing that the medians between the different timepoints of MVM infection and HU-treated cells are statistically significant.
(C) The statistical analysis has been computed with Mann Whitney Wilcoxon Test in the supporting Prism file to maintain consistency across figures. We have also added a description of this statistical analysis to the Materials and Methods section.

**P2.** The authors categorized the DNA fiber types during the course of MVM infection, observing a large fraction of new origins at 24 hpi. As MVM infected cells at 24 hpi are supposed to be in G2 cell cycle phase where no new origins are expected to fire, the authors conclude that MVM modulates origin firing.
(A) how did the authors categorize the DNA fibers into progressing, stalled, and new origin?
(B) evidence should be provided that the MVM infected cells are indeed in G2 cell cycle phase at 24 hpi

**Response:** Thank you for pointing this out. We have:

(A) Provided a new Supplemental Figure S3 where we have depicted representative replication events including (A) stalled, (B) new origin and (C) progressing replication forks. We have also schematized these replication events on the right that is used to categorize them during data analysis. These are also described in detail in the Materials and Methods section.
(B) We have previously discovered that MVM infection leads to cell cycle arrest at the G2/M border caused by transcriptional repression of the Cyclin B1 gene (Fuller et al, JVI, 2017; Adeyemi et al., PLOS Pathogens, 2014). We have provided the relevant references to the results section.

**P3.** The authors assessed the generalization of their observation by measuring the DNA fibers 24 hpi in non-synchronous U2OS cells infected with either wild-type MVM or MVM lacking NS2. NS2 is required for replication in murine but not in transformed human cells. They observed a DNA fiber shortening in either case, indicating replication stress induction at equivalent levels in U2OS cells.
(A) evidence for the lack of NS2 should be provided
(B) in the figure 1 legends, the authors write NS1-deficient mutant instead of NS2-deficient mutant (line 477)
(C) in the RPA Paper Information Fig. 1G is labeled as Fig 1I
(D) Axis label not consistent (B, C, G, H) and several data points are not within the range of the axis and are therefore not shown (compare to RPA Paper Information).

**Response:** Thank you for the suggestions. Our responses are below:

(A) We have performed western blots showing lack of NS2 after 24 hours of infection with the MVM^ΔNS2^ virus, shown in Supplemental Figure S4 and is consistent with previously published reports (Naeger et al., 1990).
(B) Thank you for identifying this typo. We have corrected it in the manuscript.
(C) Thank you for identifying this typo. We have corrected Fig. 1I to Fig. 1G in the resubmission.
(D) Thank you for identifying these missed datapoints. We have re-plotted all these graphs to show all datapoints and made the axis ranges consistent within each figure.

**P4.** Treating MVM infected cells with an CDC7 inhibitor resulted in less DNA fiber shortening (significant for infected samples but not compared to mock) and in a decrease of new origins (Fig. 2B – 2D). Moreover, treating the MVM infected cells with the CDC7 inhibitor led to reduced levels of γH2AX (indicating less replication stress) and NS1 (less MVM replication; also seen in Fig. 2F; no mock-infection shown).
(A) efficient synchronization is not shown in the data
(B) Fig. 2B: not all data points are within the range of the axis and are therefore not shown (compare to RPA Paper Information Fig 2B.pzfx). 6 data points are outside the axis limit.
Fig. 2C: not all data points are within the range of the axis and are therefore not shown (compare to RPA Paper Information Fig 2C.pzfx). 8 data points are outside the axis limit.
(C) axis label not consistent (B, C).

**Response:** Thank you for the suggestions. Our responses are below:

(A) We have performed cell synchronization studies by flow cytometry showing A9 cells are synchronized by isoleucine deprivation and enter S phase upon release into complete media at 12 hpi. These are shown in Supplemental Figure S1. This synchronization protocol has been extensively used for MVM infection studies in A9 cells, as previously published (Adeyemi and Pintel, JVI, 2012; Fuller et al., JVI, 2017; Bunke et al., JVI, 2023).
(B) Thank you for identifying these missed datapoints. We have re-plotted all these graphs to show all datapoints and made the axis ranges consistent within the figure.
(C) Thank you for identifying these inconsistencies. We have re-plotted the graphs to keep axis labels consistent.

**P5.** Ectopically expressed MVM non-structural proteins NS1 and NS2
(A) were the cells synchronized for this experiment?
(B) is mock the pUC18 transfected sample?
(C) Fig. 3C: not all data points are within the range of the axis and are therefore not shown (compare to RPA Paper Information Fig 3C.pzfx). 1 data point is outside the axis limit.
(D) is there a synergistic effect of NS1 and NS2?

**Response:** Thank you for the suggestions. Our responses are detailed below:

(A) The cells were synchronized by Isoleucine deprivation for 36 hours before being transfected with the NS1/NS2 expression vectors. The cells were released into complete DMEM media at 42 hours and processed for DFA at 24 hours post-transfection. This was compared with cells that were infected with MVM for 24 hours. We have updated Figure 3A to reflect the experimental timeline and its corresponding description in the figure legend.
(B) Yes, the sample labelled “Mock” is transfected with equivalent amount of pUC18 vector as the NS1/NS2 expression constructs that had been transfected into the cells. We have clarified this in the figure legend.
(C) Thank you for identifying these missed datapoints. We have re-plotted all these graphs to show all datapoints and made the axis ranges consistent within the figure.
(D) This is an excellent question. We have exciting new preliminary data showing that vectors co-expressing NS1 and NS2 exhibit attenuation of replication stress at 24 hours post-transfection. NS2 transcripts are produced by alternative splicing of NS1, but splicing efficiency varies between infection and transfection conditions. It has been established that cellular replication stress (such as that induced by viral proteins) impacts RNA processing in host cells. We predict that these observations are connected to R-loop stability and cellular innate immune sensing, since NS2 is readily shuttled out of the nucleus. These findings are being followed up for future studies.

**P6.** As replication stress was observable already early during infection (12 hpi) and prior to detectable NS1 protein expression, the authors concluded that NS1 alone might not be sufficient for MVM-mediated replication stress at early time points of infection and therefore assessed whether non-replicating MVM genomes lead to replication stress.
(A) Fig. 4C: not all data points are within the range of the axis and are therefore not shown (compare to RPA Paper Information Fig 4C.pzfx). 1 data point is outside the axis limit.
(B) Axis label not consistent (B, C)
(C) efficient UV-inactivation not confirmed (e.g., on protein level)
(D) UV-inactivated MVM is not supposed to induce γH2AX → based on ref.
(E) efficient synchronization is not shown
(F) the authors state that UV-MVM genome is largely made up of ssDNA. This should be shown at 12/18 hpi?
(G) is the result in Fig. 4E statistically significant?
(H) DFA analysis was performed at 18 hpi. In contrast, ChIP-qPCR was performed at 24 hpi and IF analysis at 16 hpi. Why did the authors use different times of infection?
(I) line 526-528: At 24 hours post-transfection,….. → There is no transfection in this experiment.

**Response:** Thank you for the suggestions. Our responses are detailed below:

(A) Thank you for identifying these missed datapoints. We have re-plotted all these graphs to show all datapoints in all graphs.
(B) Thank you for identifying these inconsistencies in labelling of the axes. We have re-plotted all these graphs to maintain consistency within the figure.
(C) We have provided a western blot showing UV-MVM infection does not express NS1 in Supplemental Figure S5A.
(D) We have provided a gamma H2AX western blot showing UV-MVM does not induce gH2AX upon infection of A9 cells (Supplemental Figure S5A, gamma H2AX immunoblot, along with quantifications), consistent with previously published findings (Adeyemi et al., PLOS Pathogens, 2010).
(E) We have performed cell synchronization studies by flow cytometry showing A9 cells are synchronized by isoleucine deprivation and enter S phase upon release into complete media at 12 hpi. These are shown in Supplemental Figure S1.
(F) We have provided a Taqman qPCR data showing UV-MVM infection is made up of single-stranded DNA at 18 hpi (Supplemental Figure S5B), and has also been previously published (Adeyemi et al., PLOS Pathogens, 2010).
(G) Yes. We have repeated these experiments at 18 hpi and performed statistical analysis that is presented in Fig. 4E.
(H) We have re-done these assays at 18 hpi to maintain consistency. Thank you for the suggestion.
(I) There is no transfection. It is 18 hpi post-infection. Thank you for pointing out the inconsistency.

**P7.** To assess whether RPA depletion results in MVM-induced replication stress, the authors overexpress pRPA, thereby showing that this can rescue the DNA fiber shortening upon UV-MVM infection.
(A) Fig. 5B: not all data points are within the range of the axis and are therefore not shown (compare to RPA Paper Information Fig 5B.pzfx). 16 data points are outside the axis limit.
(B) is Mock ctrl. transfected?
(C) is transfection itself the reason for the fiber shortening in the pRPA transfection? Missing comparison to ctrl. transfection.
(D) Fig. 5C: not all data points are within the range of the axis and are therefore not shown (compare to RPA Paper Information Fig 5C.pzfx). 4 data points are outside the axis limit.
(E) axis label not consistent (B, C)

**Response:** Thank you for the suggestions. Our responses are detailed below:

(A) Thank you for identifying these missed datapoints. We have re-plotted all these graphs to show all datapoints in all graphs.
(B) Yes, the sample labelled “Mock” is transfected with equivalent amount of pUC18 vector as the pRPA expression construct that had been transfected into the cells. We have clarified this in the figure legend.
(C) This is an astute suggestion. However, comparing the IdU/CldU lengths for pUC18-transfected cells with that of pRPA showed a minor statistically significant difference. We have provided the requested statistical comparison to the control transfection.
(D) Thank you for identifying these missed datapoints. We have re-plotted all these graphs to show all datapoints in all graphs.
(E) Thank you for identifying these inconsistencies in labelling of the axes. We have re-plotted all these graphs to maintain consistency within the figure.

**REVIEWER 2**

We thank Reviewer 2 for their input and suggestions for additional experiments. We have completed these experiments and our findings have strengthened the overall conclusion and validity of our findings. Their specific concerns are addressed below.

**P1.** Fig. 4F. The data would be more convincing after quantitative colocalization or closeness**.**

**Response:** Thank you for the suggestion. We have provided quantitative data of NS1, phospho-RPA and total RPA colocalization across a transverse section of the nucleus in Fig. 4F, bottom. The profile of fluorescence intensity matches between the three channels suggesting that the proteins are in the same relative location within the nuclear compartment.

**P2.** Analyses. At least instead of the term colocalization use the phrase localized within (line:227.)

**Response:** Thank you for the suggestion. We have made the corresponding change.

**P3.** Figures with western blots. Please add quantitative analyses of WBs.

**Response:** We have provided quantitative analyses for all western blots and these results support our conclusions. We have described how this analysis was performed in the Materials and Methods section.

**REVIEWER 3**

**P1.** The current version of the manuscript lacks important details about how the DNA fibre analyses were done, thereby preventing the interpretation of the data and the conclusions made by the authors. The authors present data potentially supporting a role of the viral genome, the viral protein NS1 and NS2, in promoting replication stress. Without proper control experiments and further characterization of these phenotypes, the current data do not support the conclusions made by the authors.

**Response:** Thank you for pointing out this weakness. We have included a more detailed protocol regarding DFA analyses in our Materials and Methods, as well as a new Supplemental Figure S3 where we have schematized the interpretation and scoring criterion to support our conclusions.

**P2.** Provide a detailed protocol on how the DNA fibre assays were quantified and images for the phenotype considered stalled replication forks vs new origin firing.

**Response:** We have added extra detail clarifying how DFAs are quantified, as well as providing a diagram depicting examples of stalled, progressing, and new origin forks and diagrams clarifying each of these in Supplemental Figure S3. 

**P3.** In figure 1E, the analysis of the phosphorylation of H2AX by western blot is not robust (as shown by the major difference between γ-H2Ax levels obtained in Fig.1E vs Fig. 2E) γ-H2Ax levels should be evaluated by immunofluorescence, which is more sensitive and more robust. Co-investigation of γ-H2Ax levels and pRPA would also be essential to conclude that DNA damage signalling is induced after replication stress in MVM-infected cells.

**Response:** Thank you for this suggestion. We have provided co-immunofluorescence of γH2AX with NS1/total RPA in Supplemental Figure S2 and pRPA with NS1/total RPA in Supplemental Figure S6. We have also provided quantification of the γ-H2AX western blot that shows the stepwise increase in cellular DDR in Fig. 1E. These studies revealed that γH2AX signals are not detectable at robust levels until 18 hpi (Supplemental Figure S2, lane 1 compared to 2) whereas there are robust levels of phospho-RPA colocalized with NS1 at 12 hpi (Supplemental Figure S6, lane 1). We have provided a description of this in the relevant Results sections.

**P4.** It is complicated to investigate if the replicative MVM genome also acts as a decoy for RPA. However, validating the hypothesis that ssDNA is leading to this phenotype can be done by investigating if the transfection of scrambled ssDNA also led to the same phenotype or if an MVM genome containing a mutation in the NS1 gene (should also inactivate replication) led to a similar phenotype.

**Response:** Thank you for the suggestion. To support our conclusion that ssDNA is sufficient to induce cellular replication stress, we have designed small oligos of the P4 region on the MVM genome, transfected them into A9 cells during parasynchronization, and performed DFAs on these cells. These exciting new findings are presented in Fig. 4G, revealing that the single-stranded P4 sequence is sufficient to induce replication stress. This data validates our overall model and opens up exciting new opportunities to identify open chromatin regions on viral genomes using ATAC-seq and determining their ability to sequester cellular RPA.

**P5.** Cytometry analysis of the cell cycle should be provided to show that isoleucine depletion lead to synchronization of cells in G0/G1 and cell cycle progression in S and G2 phases after the addition of complete media.

**Response:** Thank you for this suggestion. We have provided FACs analysis of our cells after synchronization and release in Supplemental Figure S1.

**P6.** In Fig 2, the authors should show that CDC7 is inhibited in iCDC7-treated cells. This could be done by doing a western blot against CDC7 or phosphorylated MCM2 as described previously (Montagnoli et al. (2008) Nature Chemical biology).

**Response:** Thank you for this suggestion. We have provided a western blot (Fig. 2E) and immunofluorescence images (Fig. 2F) for pMCM2 in iCDC7 treated samples. These findings confirm that the CDC7 inhibitor is functional and modulate MVM replication. 

**P7.** Validation of MVM genome inactivation should be provided.

**Response:** We have provided validation that UV-MVM is inactivated by western blot for NS1 in Supplemental Figure S5A, where infection with the UV-inactivated virus did not produce NS1 (immunoblot) and did not generate the plus strand indicative of replication (monitored by Taqman qPCR in Supplemental Figure S5B).

***Minor Comments***

**REVIEWER 2**

Was there any specific reason why the IdU was not introduced prior to infection?
The colors used in the figures might not be visible for people with color vision deficiencies, I would suggest changing the color schemes in the figures.

**Response:** Thank you for the suggestion. The pulsing of IdU prior to infection followed by CldU pulse prior to harvest is used to study the mechanisms of DNA replication fork processing. These experiments are the focus of future studies investigating how MVM-induced host-cell perturbations modulate DNA end resection.

We have modified the color schemes for the fiber categorization in order to make it more accessible.

1) The last sentence in the abstract should be rewritten and clarified.

**Response:** We have rewritten and clarified the last sentence.

2) Line 74: nuclear export

**Response:** Changed

3) Line 86: jumpstart ;)

**Response:** Thank you. Trying something new. We have changed it to “initiate”.

4) Figure 1A/B: is there a statistically significant change between mock and 12 and 18 hpi and
HU. Please show the analyses in the Figure. Fig. 1E Tublin = tubulin.

**Response:** We have shown complete statistical analysis in the revised Fig. 1B/C between mock, 12hpi, 18hpi, 24hpi and HU. We have changed Tublin to Tubulin.

5) Fig 1G and 1H: Is the effect of NS2-expression mutant infection on replication fiber progress
(stalling and emergence of new origins) similar to that of wt MVM infection?

**Response:** As shown in Supplemental Figure S4B, the NS2 expression mutant of MVM has a similar impact on replication fork categories as wild-type MVM. We also discuss this data in the relevant results section.

6) Add spaces between the result chapters

**Response:** Done

7) Fig 3 B/C. It would have been very interesting to see if there is a statistically significant
difference between wt MVM and ectopic expression of NS1 and NS2 (if that is possible to
include in the figure).

**Response:** We have provided the requested statistics in the revised Fig. 3, finding that there is a significant difference between ectopic expression and infection of MVM at 24 hpi.

8) Line 208. Could you please explain more carefully what is the time schedule of the MVM
NS1 expression.

**Response:** We have provided a clarification of this section in the results.

9) Lines: 182-184 and 240-241 Can this be said merely based on the increase of NS1?

**Response:** Since NS1 is critical for MVM replication, the expression and replication of parvovirus genomes are coupled. This occurs through a rolling hairpin replication process. Therefore, for parvoviruses, NS1 is an established marker for autonomous parvovirus replication, as previously published (Willwand et al., JBC, 1998).

**REVIEWER 3**

The concept of DDR vs DNA damage signalling and repair should be revisited in the whole manuscript. DNA damage response (DDR) refers to the signalling induced to stop the progression of the cell cycle upon detection of DNA damage.

**Response:** Thank you for the suggestion. We have revisited all mentions of DDR in the manuscript to make them distinct from DNA damage signaling.

Detailed drug treatments (HU, iCDC7) should be added to the manuscript.

**Response:** We have provided details on the CDC7 inhibitor and HU treatment conditions in the Materials and Methods.

In summary, we have: (i) included new experimental data to address the major scientific concerns raised by reviewers, (ii) carefully addressed each of the evaluative comments provided by the reviewers, and (iii) revised the text to further elucidate our findings. We believe these revisions have greatly improved the manuscript and thank the reviewers for their helpful comments. We hope that the revised manuscript is suitable for publication in *PLOS Pathogens*.

Sincerely

Kinjal Majumder, PhD

Assistant Professor of Oncology and Molecular Virology
McArdle Laboratory for Cancer Research
Virology Program, UW Carbone Cancer Center
Institute for Molecular Virology
[kmajumder@wisc.edu](mailto:kmajumder@wisc.edu)
